# Identification of key genes associated with endometriosis and endometrial cancer by bioinformatics analysis

**DOI:** 10.3389/fonc.2024.1387860

**Published:** 2024-11-22

**Authors:** Ruyue Ma, Yu Zheng, Jianing Wang, Hong Xu, Ruirui Zhang, Zhijia Xie, Lei Zhang, Ruiheng Zhao

**Affiliations:** Department of Obstetrics and Gynecology, Suzhou Ninth People’s Hospital, Suzhou Ninth Hospital Affiliated to Soochow University, Suzhou, China

**Keywords:** bioinformatics, biomarker, gene expression, endometriosis, endometrial cancer

## Abstract

**Background:**

Endometriosis (EMS) is acknowledged as a risk factor for the development of endometrial cancer (EC), although the precise molecular mechanisms that underpin this association have yet to be fully elucidated. The primary objective of this investigation is to harness bioinformatics methodologies to identify pivotal genes and pathways that may be implicated in both EMS and EC, potentially offering novel therapeutic biomarkers for the management of endometriosis.

**Methods:**

We acquired four datasets pertaining to EMS and one dataset concerning EC from the Gene Expression Omnibus (GEO) database. Differentially expressed genes (DEGs) in EMS and EC cohorts, in comparison to controls, were ascertained utilizing the limma package. Subsequently, we conducted a series of bioinformatic analyses, including Gene Ontology (GO), Kyoto Encyclopedia of Genes and Genomes (KEGG) pathway analysis, and protein-protein interaction (PPI) analysis, to delineate pathways associated with the identified DEGs.

**Results:**

Our bioinformatics analyses disclosed 141 shared DEGs between EMS and EC groups relative to the control cohort. GO analysis demonstrated that these genes are predominantly involved in the regulation of growth and development, as well as signal transduction pathways. KEGG analysis underscored the significance of these genes in relation to the JAK-STAT signaling pathway and leukocyte transendothelial migration. Furthermore, PPI analysis pinpointed ten central genes (APOE, FGF9, TIMP1, BGN, C1QB, MX1, SIGLEC1, BST2, ICAM1, MME) exhibiting high interconnectivity. Notably, the expression levels of APOE, BGN, C1QB, and BST2 were found to correlate with cancer genomic atlas data, and were implicated in tumor immune infiltration. Strikingly, only APOE and BGN demonstrated a significant correlation with patient prognosis.

**Conclusion:**

This comprehensive bioinformatics analysis has successfully identified key genes that may serve as potential biomarkers for EC. These findings significantly enhance our comprehension of the molecular underpinnings of EC pathogenesis and prognosis, and hold promise for the identification of novel drug targets.

## Introduction

Endometriosis (EMS), characterized by the presence of endometrioid tissue outside the uterine cavity, affects approximately 5% to 10% of women of reproductive age worldwide and is a leading cause of dysmenorrhea, infertility, and chronic pelvic pain (1). EMS is a benign lesion that causes pelvic tissue adhesion, forming nodules and masses, and is characterized by its dependence on sex hormones. The malignant manifestations of EMS are similar to those of tumors, with lesions that are extensive, diverse, highly invasive, and recurrent.

Endometrial cancer (EC) is the seventh most common cancer among women worldwide and is one of the most prevalent gynecological tumors (2). With an average annual growth rate of 1.9%, EC significantly impacts the quality of life for patients and places a substantial burden on healthcare resources (3). Recent studies have indicated that EC is more prevalent in individuals with EMS than in the general population, and EMS is an independent risk factor for EC prognosis. This suggests that patients with EMS may have a higher risk of developing EC (4, 5). However, the molecular mechanisms underlying the association between EMS and EC are not well understood.

Patients with EMS and EC share similar pathological mechanisms, including genomic alterations, inflammation, stem cells, immunogenicity, and estrogen dependence (5, 6). An increasing number of studies suggest that endometriosis is a systemic inflammatory disease. The concentration of cytokines in endometriosis is abnormally elevated, including IL-1β, IL-6, IL-28, tumor necrosis factor, CCL2, CCL5, and VEGF (7, 8), which are related to the progression of EMS and the development of EC (9–11). Research has found that immune cells are involved in the pathogenesis of EMS, with an increased number of lymphocytes in peritoneal fluid and a higher concentration of peritoneal macrophages. Additionally, an imbalance between Th1/Th2 cells contributes to the progression of the disease (12, 13). Therefore, we consider whether there are key genes and pathways that underlie common molecular mechanisms in the development of these two diseases. Screening differentially expressed genes (DEGs) between endometrial cancer and endometriosis may offer an alternative approach to understanding the mechanisms of carcinogenesis and recurrence in endometriosis, as well as constructing new potential serum biomarkers for the early diagnosis of EC patients.

Bioinformatics analysis integrates and analyzes biological data through various bioinformatics tools, which is one of the crucial means of life science research. To discover the pathogenesis of EMS related to EC, we collected three EMS datasets and one EC dataset from the Gene Expression Omnibus (GEO) database using various comprehensive bioinformatics tools. We identified hub genes associated with EMS in EC, predicted common molecular pathogenesis, and potential therapeutic drugs. We validated the expression patterns of hub genes in EC. Finally, we explored the immune cell characteristics of EC to reveal the association of hub genes with the immune system, which may provide a new direction for treatment.

## Materials and methods

### Data collection

In this study, data sets are from the National Center for Biotechnology Information (NCBI) pool of gene expression (GEO, https://www.ncbi.nlm.nih.gov/geo/). The data in the GEO database we used contained preprocessed and normalized data, which is suitable for direct use in downstream analysis. Three endometriosis datasets, GSE7305, GSE23339, and GSE25628, and one endometrial cancer dataset, GSE17025, were used. The GSE7305 dataset contains 10 ectopic endometrium and 10 samples of normal endometrium. GSE23339 contains 10 cases of ectopic endometrium and 9 cases of normal endometrium. GSE25628 contains 7 cases of ectopic endometrium, 9 cases of eutopic endometrium, and 6 cases of normal endometrium. GSE17025 contains 12 cases of normal endometrium and 91 cases of tumor. The downloaded data were processed using the R package limma followed by calibration, standardization, and log2 transformation, and details about the dataset are summarized in **Table 1**.

**Table 1:** Details of GEO data used in this study.

GEO numbers	Experimental group	Control group	PMID
GSE7305	10	10	17640886
GSE17025	91	12	21619611
GSE23339	10	9	21436257
GSE25628	16	6	23460397

### Analysis of differentially expressed genes

R Studio software was utilized to process and standardize the files. Four GEO datasets were preprocessed. For the genes corresponding to multiple probes, the average value was taken as the expression level of the gene, and the expression levels of all genes were converted into log2 (expression level). The “limma” R package was applied to analyze the differences between the experimental and control groups. The threshold of difference analysis was set as p-value <0.05 and FC >1.5. Overlapping DEGs from the four database screenings were utilized in subsequent GO enrichment, KEGG pathway analysis, and protein-protein interaction (PPI) analyses. We then used Venn software online (https://jvenn.toulouse.inrae.fr/app/example.html) to visualize the common DEGs.

### Gene ontology and pathway discovery based on gene enrichment analysis

Enrichment analysis of gene sets provides unique biological properties of genes, including biological processes, cellular components, and molecular functions. KEGG pathway enrichment analysis explores the critical pathways involved in disease occurrence and progression. KEGG and GO enrichment analysis of differential genes was conducted with the R package clusterProfiler, with a p-value threshold of less than 0.05. We considered the input dataset to enrich the pathway or term if it met this criterion.

### PPI network and Hub gene acquisition

The activity of protein-protein interactions (PPIs) is regarded as a significant target for cell biology research and is vital for elucidating key genes and necessary gene modules during cancer development. Common DEGs (version 11.0, https://string-db.org/) were inputted into STRING, a search tool for retrieving interacting genes, to generate PPI networks with a medium confidence score of 0.400. The STRING database comprises electronically inferred interaction data to ensure the most comprehensive coverage of potential protein interactions possible. The PPIs obtained through the analysis with Cytoscape software (https://cytoscape.org/) were visualized and analyzed using this powerful bioinformatics software. Additionally, the Cytoscape plugin CytoHubba (https://apps.cytoscape.org/apps/cytohubba) was employed to extract Hub genes, and the MCC function of CytoHubba was used to identify the top 10 Hub genes from the PPI network.

### Expression verification of Hub genes

To further select precise biomarkers, we performed Gene Expression Profiling (GEPIA, http://gepia.cancer-pku.cn/). This web-based tool provides fast and customizable functionality to display gene expression profiles from the TCGA database while performing survival analysis of genes. To explore the survival differences between different groups. Immunohistochemical (IHC) data for common genes were retrieved in the Human Protein Atlas (HPA) project, which contains detailed information about samples.

### TF-gene interactions

NetworkAnalyst (https://www.networkanalyst.ca/) was employed to identify genes, and TF has identified common genetic interactions, the research of transcription factors, and regulation networks between the target genes. NetworkAnalyst is a comprehensive network platform used to perform gene expression on many species, enabling them to be subjected to meta-analysis.TF-gene interaction network of network platform from NetworkAnalyst ENCODE (https://www.encodeproject.org/) contained in the database.The ENCODE database provides abundant information on transcription factor binding sites, which can provide strong support for the study of TF gene interactions.

### TF miRNA co-regulation network

TF miRNA co-regulation interaction refers to the complex interaction among Transcription factors, miRNAs, and their target genes. The interaction of TF miRNA co-regulation was collected from the RegNetwork reservoir. Transcription factors are proteins that can bind to specific sequences of DNA, and miRNAs are small RNA molecules that regulate the transcription and translation process of genes by binding to the miRNA of target genes, thereby affecting the expression level of genes. NetworkAnalyst was used to visualize the TF miRNA co-regulation network.

### Immune infiltration analysis of hub genes

Tumor immunity infiltration online database (TIMER, https://cistrome.shinyapps.io/timer/) is applied to analyze different types of cancer immunity infiltration. TIMER can analyze the relationship between the cancer immune cell infiltration level and hub genes. The relationship between somatic copy number alterations (SCNA) of potential prognostic hub genes and infiltrating immune cells was explored through related modules. A cutoff value of P < 0.05 was applied as the threshold.

### Identification of candidate drugs

Drug molecular identification is a key component of research, and the drug-Gene Interaction Database (DGIdb, http://www.dgidb.org) is a web-based resource that weaves the genome of druggable genomes into known drug interactions and potential druggable targets. We input the co-expressed DEGs and evaluate the DEGs in DGIdb to find potential therapeutic drugs.

### RNA isolation and qRT-PCR

The human endometrial cancer cell line (HEC - 1B and Ishikawa) and the normal endometrial epithelial cell line (HEEC) were both purchased from ATCC. These cell lines were cultured in a culture flask containing DMEM medium supplemented with 10% fetal bovine serum and 1% penicillin-streptomycin in an incubator set at 37°C with 5% CO_2_. Cells from the endometrial cancer cell lines and the normal endometrial cell line, both in the logarithmic growth phase, were collected separately. Total RNA was isolated from the cells following the manufacturer’s instructions using the PureLink RNA Mini Kit (Invitrogen, Carlsbad, CA, USA), and the RNA was reverse transcribed using the PrimeScript RT Kit with gDNA Eraser (Takara, Dalian, China). Subsequently, RT-qPCR amplification was conducted using PowerUp SYBR Green Master Mix (Thermo) according to the manufacturer’s protocol, with GAPDH serving as the reference gene. The PCR protocol consisted of an initial denaturation at 95°C for 15 seconds followed by 40 cycles at 95°C for 5 seconds, and 60°C for 1 minute. The primer sequences are presented in **Table 2**. Each sample was prepared in triplicate.

**Table 2:** The primers used for RT-qPCR.

Target gene	Forward primer	Reverse primer
AOPE	5′-GTTGCTGGTCAC ATTCCTGG-3′	5′-GCAGGTAATC CCAAAAGCGAC -3′
BGN	5′-TGACTGGCATC CCCAAAGAC-3′	5′-GAGTAGCGAA GCAGGTCCTC-3′
BST1	5′-GGAGGAGCTTG AGGGAGAG-3′	5′-CTCAGTCGCT CCACCTCTG-3′
C1QB	5′-GCTCCTGGGCCT AATCGATA-3′	5′-GCCGACTTTTCC TGGATTCC-3′
GAPDH	5′-TCCTCTGACTTCAAC AGCGACAC-3′	5′-CACCCTGTTGCTGT AGCCAAATTC-3′

## Results

### Identification of DEGs and common genes between EMS and EC

**Figure 1:**
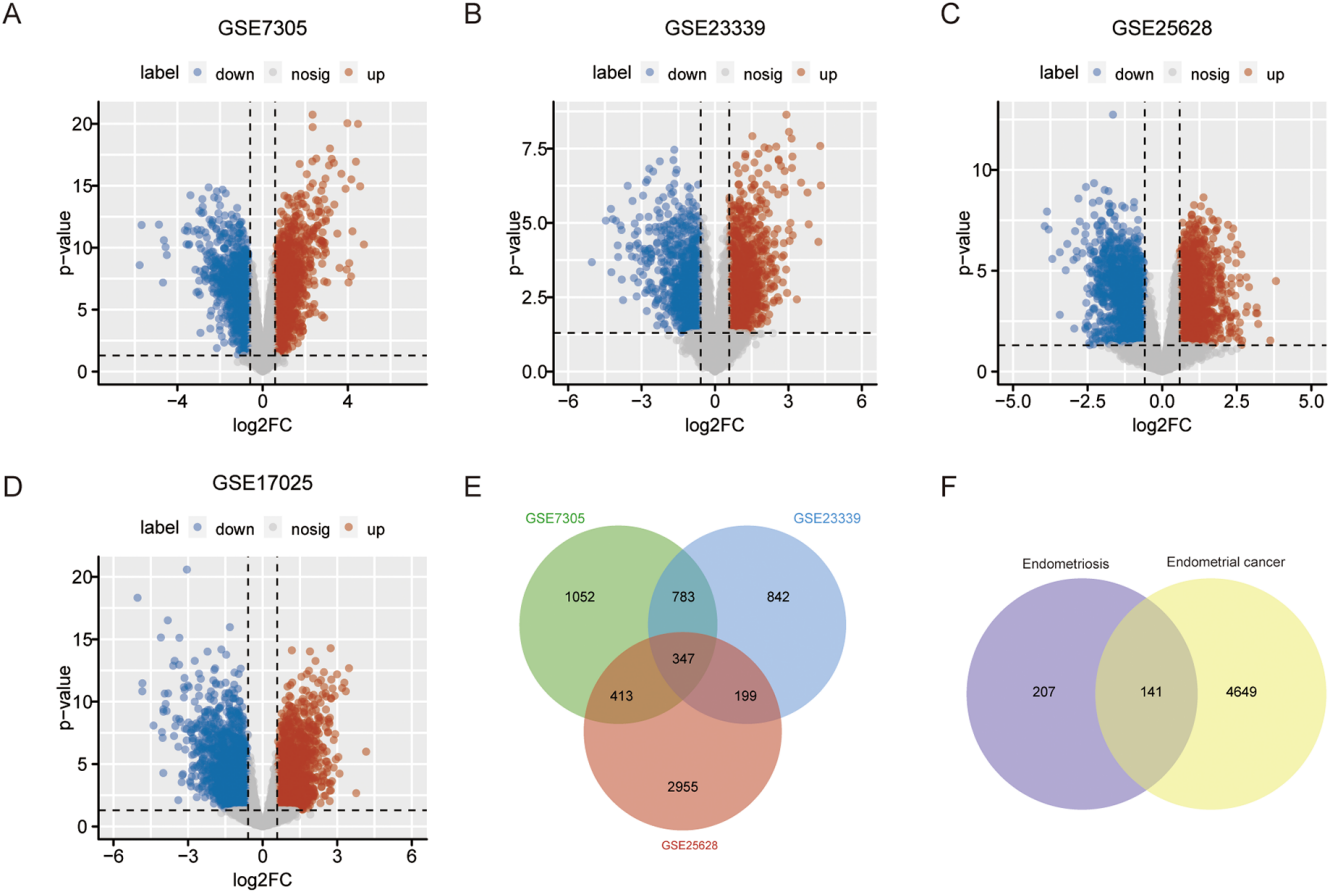
Venn diagram and Volcano diagram of DEGs in the Endometriosis and Endometrial Cancer Microarray data set. DEGs in the volcano plot shown in GSE7305 **(A)**, GSE2339 **(B)**, GSE25628 **(C)**, and GSE17025 **(D)** refer to genes with P values < 0.05 and [logFC] > 1.5. Red dots indicate genes upregulated in the experimental group relative to the control group, and blue dots indicate genes downregulated in the experimental group relative to the control group. **(E)**, Venn diagram of intersection of DEGs from three endometrial transposition datasets. **(F)**, Venn diagram of differential genes in endometriosis and endometrial cancer.

**Figure 2:**
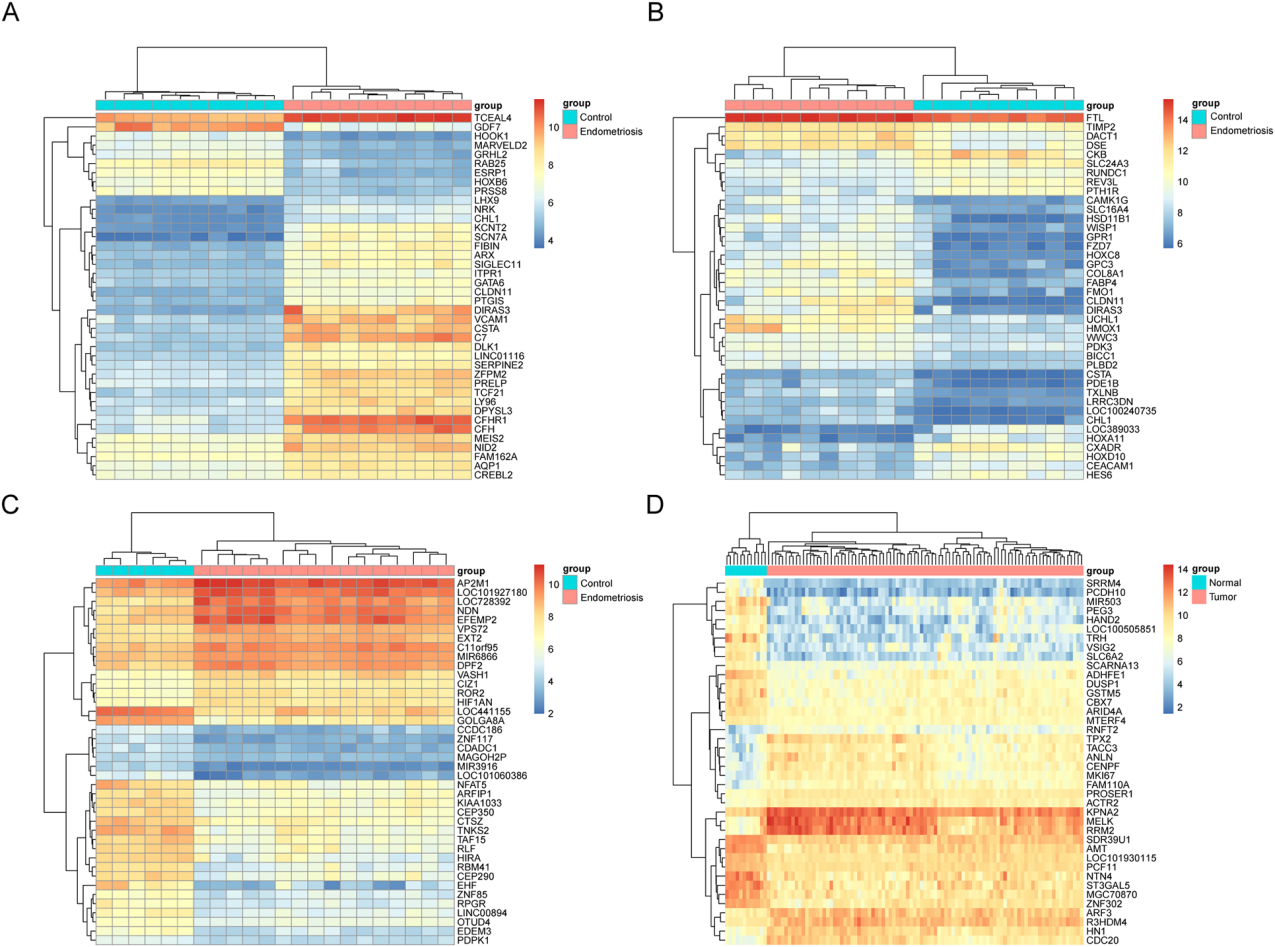
Heat map of the top 40 upregulated and downregulated degs in the microarray dataset. Top 40 DEGs in microarray datasets GSE7305 **(A)**, GSE2339 **(B)**, GSE25628 **(C)**, and GSE17025 **(D)**, DEGs refer to genes with P values <0.05 and Log[FC]>1.5. Red indicates upregulation and blue indicates downregulation. Each column represents a GEO ID, and each row represents a gene name.

All four datasets were analyzed for gene expression using the R limma package, and genes with P values <0.05 and Log [FC]>1.5 were designated as DEGs. As depicted in the volcano plot of **Figure 1**, specific information about the four datasets is summarized in **Table 1**. In the GSE7305 dataset, 2595 DEGs were identified, including 1278 upregulated and 1317 downregulated genes. In GSE2339, 2171 DEGs were identified, with 1016 upregulated and 1155 downregulated genes. In GSE25628, 3842 DEGs were identified, comprising 1819 upregulated and 2023 downregulated genes. In GSE17025, 4790 DEGs were identified, including 2246 upregulated and 2544 downregulated genes (**Table 3**). The DEGs for each dataset were visualized using volcano plots (**Figures 1A–D**). After standardization, we identified 141 differentially expressed genes (**Figure 1E**) by comparing the DEGs present across the four datasets, with 56 differentially expressed genes being consistent. Among these, 33 were upregulated and 23 were downregulated (**Table 4**). The top 40 DEGs from all four datasets were visualized on a heat map (**Figures 2A–D**).

**Table 3:** The number of up-regulated and down-regulated genes and the number of differentially expressed genes in the differential gene analysis of each expression profile.

GEO No.	Upregulated genes	Downregulated genes	Differential genes
GSE7305	1278	1317	2595
GSE17025	2246	2544	4790
GSE23339	1016	1155	2171
GSE25628	1819	2023	3842

**Table 4:** DEGs co-expressed in the four data sets.

Category	Gene symbol
Upregulted	BST2 RPP25 HOXC6 FHL2 PAPSS2 CDH3 TIMP1 IL4R CHST15 RNASE1 QSOX1 PLTP HS3ST3A1 ST6GALNAC5 GGT5 BGN ENO2 SIGLEC1 RNASE6 ICAM1 NCF4 SPSB1 HCLS1 MX1 ADAMTSL2 ENC1 APOE OSMR C1QB TNFAIP3 SULF1 THBD PIM1
Downregulated	GABRP P2RY14 MAP3K1 REV3L ARSJ COBL MME ACSL CTNNA2 TOM1L1 HEY2 GRAMD1C SEMA5A CWH43 IL20RA TRH PGGHG ADGRG2 CRYZ ANXA3 FAM189A2 SEMA3E FGF9
Inconsistency	ESRP1 NXN PAMR1 CLDN7 CD248 TFCP2L1 RAMP1 CD24 DSP HN1L PRRG4 TSPAN13 MAP7 STIL NPR2 SCARA3 PRR15L PLS1 SPAG1 HYOU1 MYO6 TMEM158 FZD5 SFN SLC25A1 MYLIP CDK2AP2 FBLN1 KRT18 KLF5 PRKX TCN1 TCF21 GATA6 PRELP TCEAL2 BNC2 PDLIM3 ITM2A PLN FBXL7 JADE2 KLF2 PDGFRL ROBO3 NEK7 TAGLN FMOD ASAP3 CLDN5 CYBRD1 TSPAN4 EFEMP1 WISP1 PLOD2 CTSF RARRES2 LIMS2 OLFML1 ACTA2 THBS4 MYL9 DCN NSG1SERPINF1 CMAHP ABI3BP COLEC12 ZNF395 EMILIN1 THBS1 COL16A1 JAM2 GLT8D2 CPQ CXCL12 MORC3 ACOX2 BST1 GFPT2 CNTN1 NR4A1 MFAP4 ADAMDEC1 GADD45B

### Enrichment analysis of differential genes

**Figure 3:**
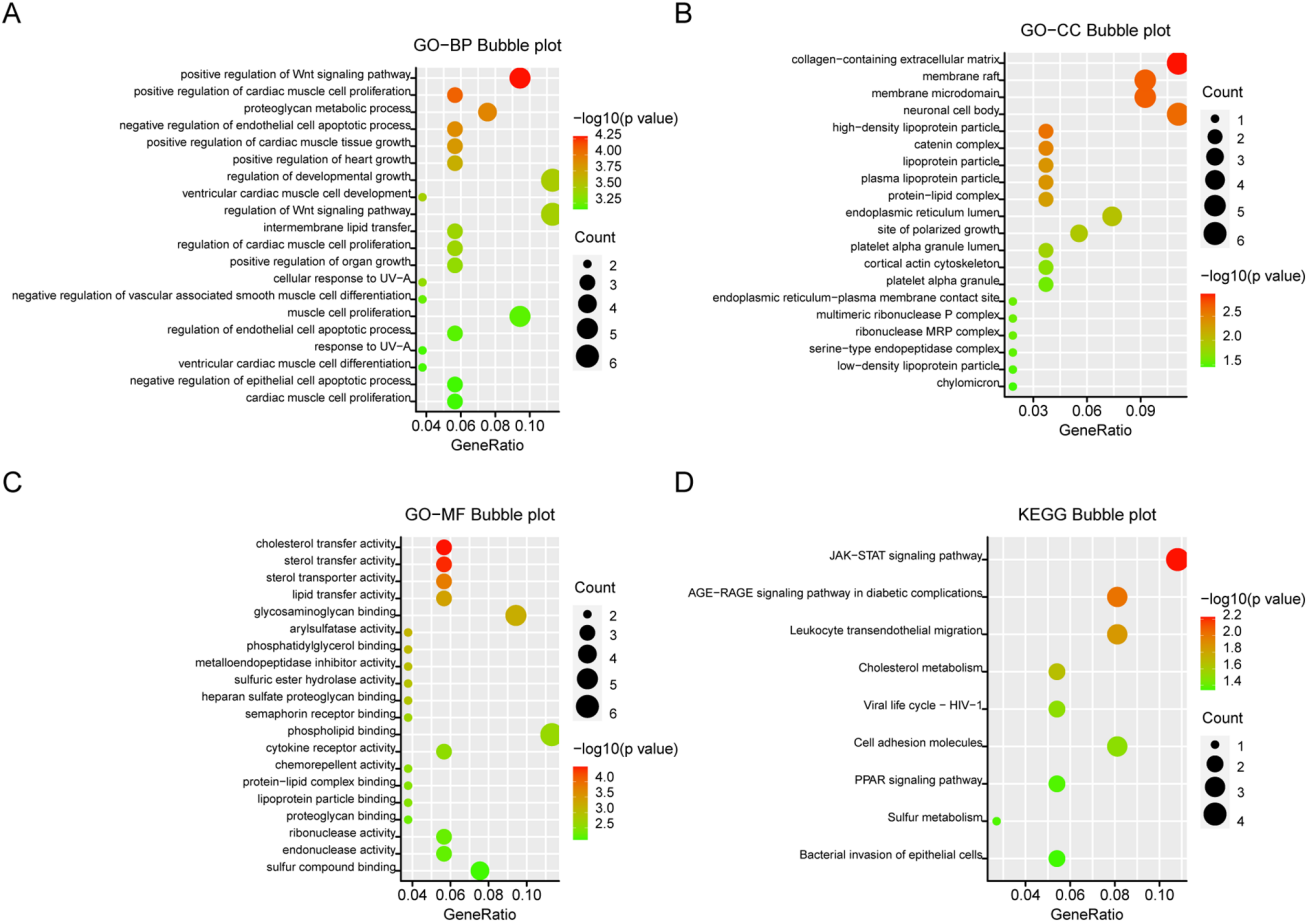
GO and KEGG analysis of DEGs. **(A)** Biological aspects of the process. **(B)** cellular components. **(C)** Molecular function. **(D)** KEGG pathways. The Y-axis shows the enriched categories and the X-axis shows the enrichment scores. Bubble size indicates the number of genes located in functional regions.

KEGG and GO analyses were conducted on the 141 DEGs to explore the biological functions of the integrated DEGs. In Biological Process (BP), DEGs were significantly enriched in processes related to growth and development, regulation of the Wnt signaling pathway, and proteoglycan metabolism (**Figure 3A**). In Cellular Component (CC), DEGs were primarily involved in the collagen-rich extracellular matrix, neuronal cell bodies, and membrane structures (**Figure 3B**). Molecular Function (MF) analysis revealed significant enrichment in glycosaminoglycan binding, cholesterol, and lipid transfer activities (**Figure 3C**). KEGG pathway analysis indicated that DEGs were mainly enriched in the JAK-STAT signaling pathway and leukocyte migration through endothelial cells (**Figure 3D**). Significantly enriched terms were displayed in bubble plots using the R package, with a criterion of *P <*0.05.

### PPI network construction and hub gene selection

**Figure 4:**
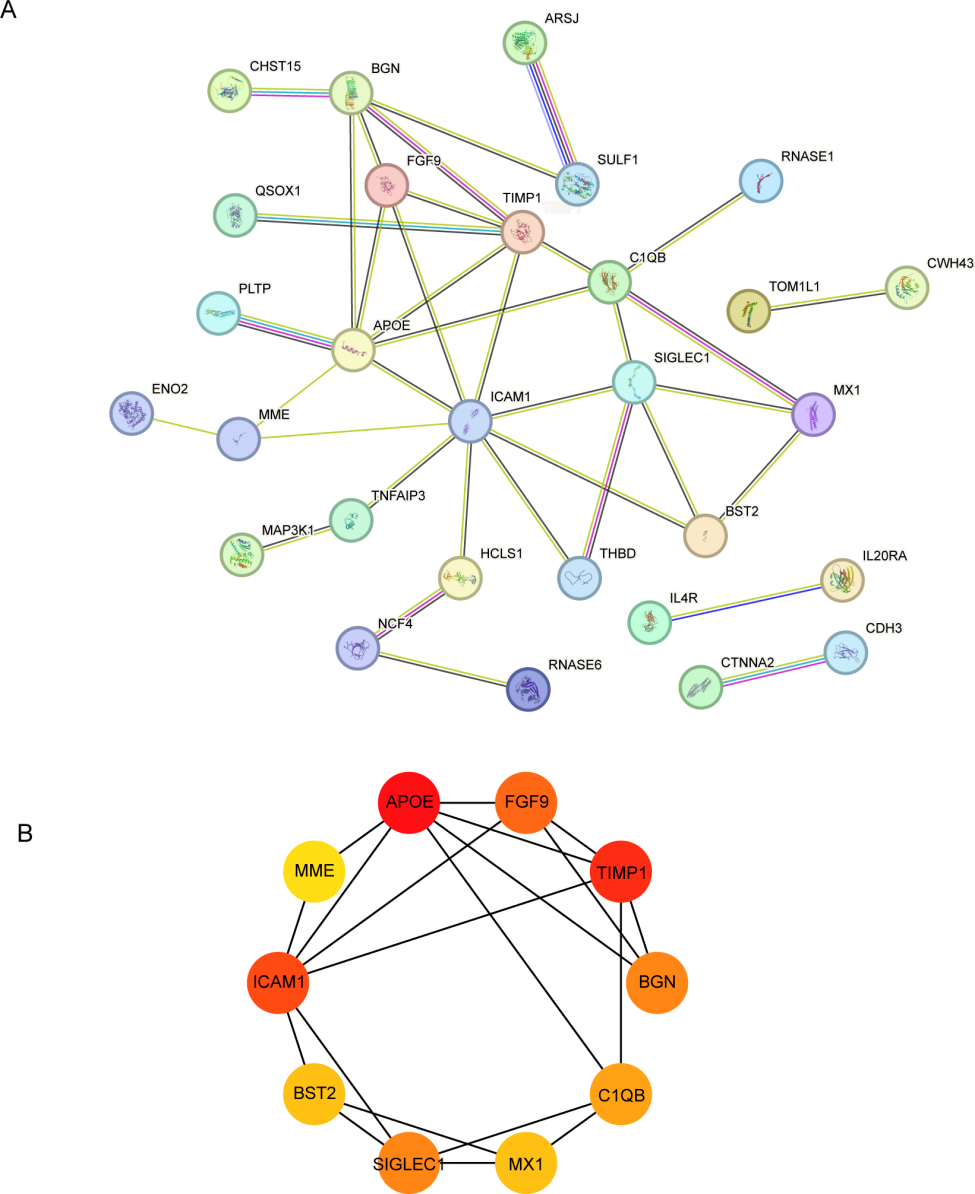
PPI network of DEGs and interaction network of top 10 hub genes. **(A)** PPI network of hub genes constructed from the STRING online database. **(B)** the top 10 hub genes were examined from the PPIs network of common differentially expressed genes, and based on the topological analysis of these 10 genes, the degree of ICAM1, APOE and TIMP1 was 7,6,5.

The 56 DEGs were uploaded to the STRING database (https://string-db.org/cgi/) with a confidence score threshold of >0.4, and the resulting PPI network was visualized using Cytoscape software. The PPI network comprised 56 nodes and 17 edges (**Figure 4A**), and the CytoHubba plugin identified the hub genes within the PPI network. Using the MCC algorithm for sorting, the top 10 hub genes selected were APOE, FGF9, TIMP1, BGN, C1QB, MX1, SIGLEC1, BST2, ICAM1, and MME (**Figure 4B**).

### TF-gene interaction and TF-miRNA co-regulatory network

**Figure 5:**
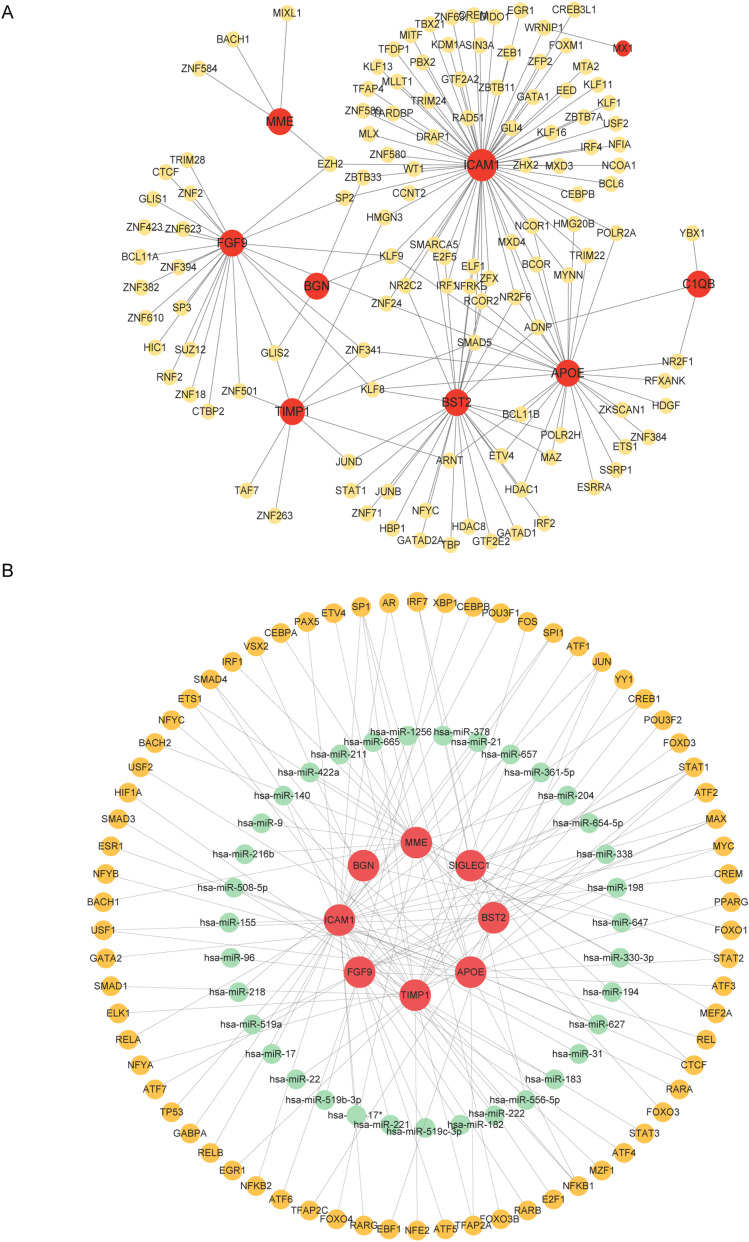
Network of TF genes interacting with common DEGs and TF-miRNA co-regulatory network. **(A)** Highlighted red nodes represent common genes and other nodes represent tf genes. **(B)** The TF-miRNA co-regulatory network consists of 235 nodes and 307 pieces, which include 70 TF-genes, 37 miRNAs, and 8 DEGs. The red color is the DEGs, the green node represents the miRNA, and the other nodes represent the TF-genes.

TF-gene interactions were collected using NetworkAnalyst. For the hub genes (APOE, FGF9, TIMP1, BGN, C1QB, MX1, SIGLEC1, BST2, ICAM1, MME), TF-gene interactions were analyzed. The interactions between TFs and common DEGs are shown in **Figure 5A**. The network contains 130 nodes and 171 edges. Among them, ICAM1 is regulated by 69 TFs, and APOE and BST2 are each regulated by 29 TFs. These TFs regulate more than one interaction in the network with a common DEG. TF-miRNA co-regulatory network analysis reveals the interactions of miRNAs and TFs with common DEGs, which may be responsible for regulating the expression of DEGs. The network created by the TF-miRNA co-regulatory analysis consisted of 235 nodes and 307 edges. **Figure 5B** shows the co-regulatory network of TF-miRNAs.

### Validation of hub genes in the TCGA database

**Figure 6:**
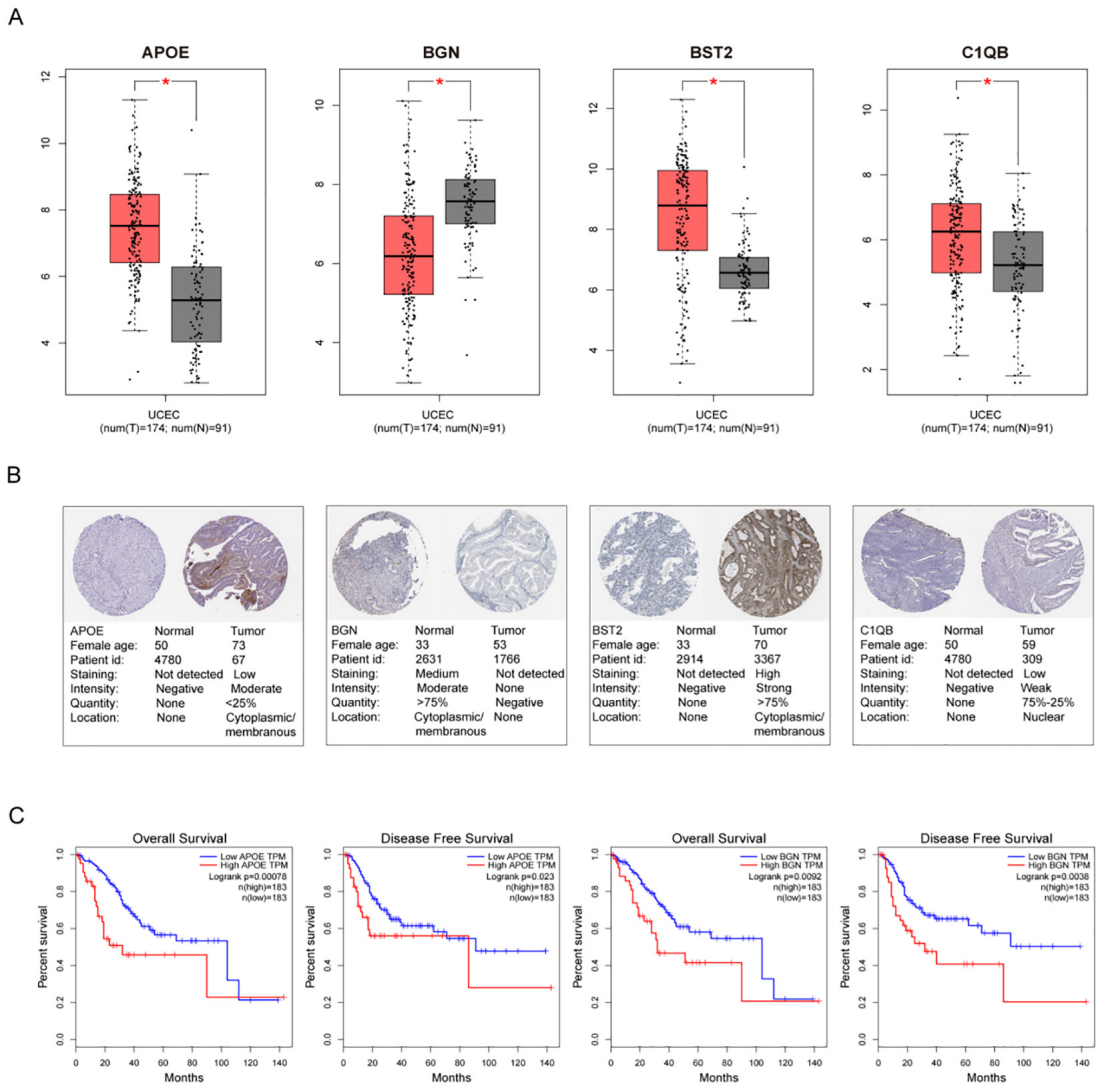
Gene Alterations and Protein Expression of hub Genes in TCGA. **(A)** The endometrial cancer database of GEPIA showed significant expression of 4 DEGs, compared with normal endometrial tissues, respectively. **(B)** Validation of hub genes by Human Protein Atlas database **(C)** Significant OS and DFS analysis of APOE and BGN between high and low expression on GEPIA.

The top 10 hub genes were used for subsequent analyses. The GEPIA database was used to investigate differences in gene expression levels between early tumor tissues and normal tissues (**Figure 6A**). The results showed that four hub genes, APOE, BGN, BST2, and C1QB, were abnormally differentially expressed in normal tissues and endometrial cancer. Additionally, the immunohistochemical (IHC) staining extracted from the Human Protein Atlas Database (HPA) also showed differential protein expression levels of hub genes, as shown in **Figure 6B**. Among the 10 hub genes, only the down-regulated genes APOE and BGN were associated with EC prognosis, confirming the reliability of the hub genes we identified (**Figure 6C**).

### Immune infiltration analysis of hub genes

**Figure 7:**
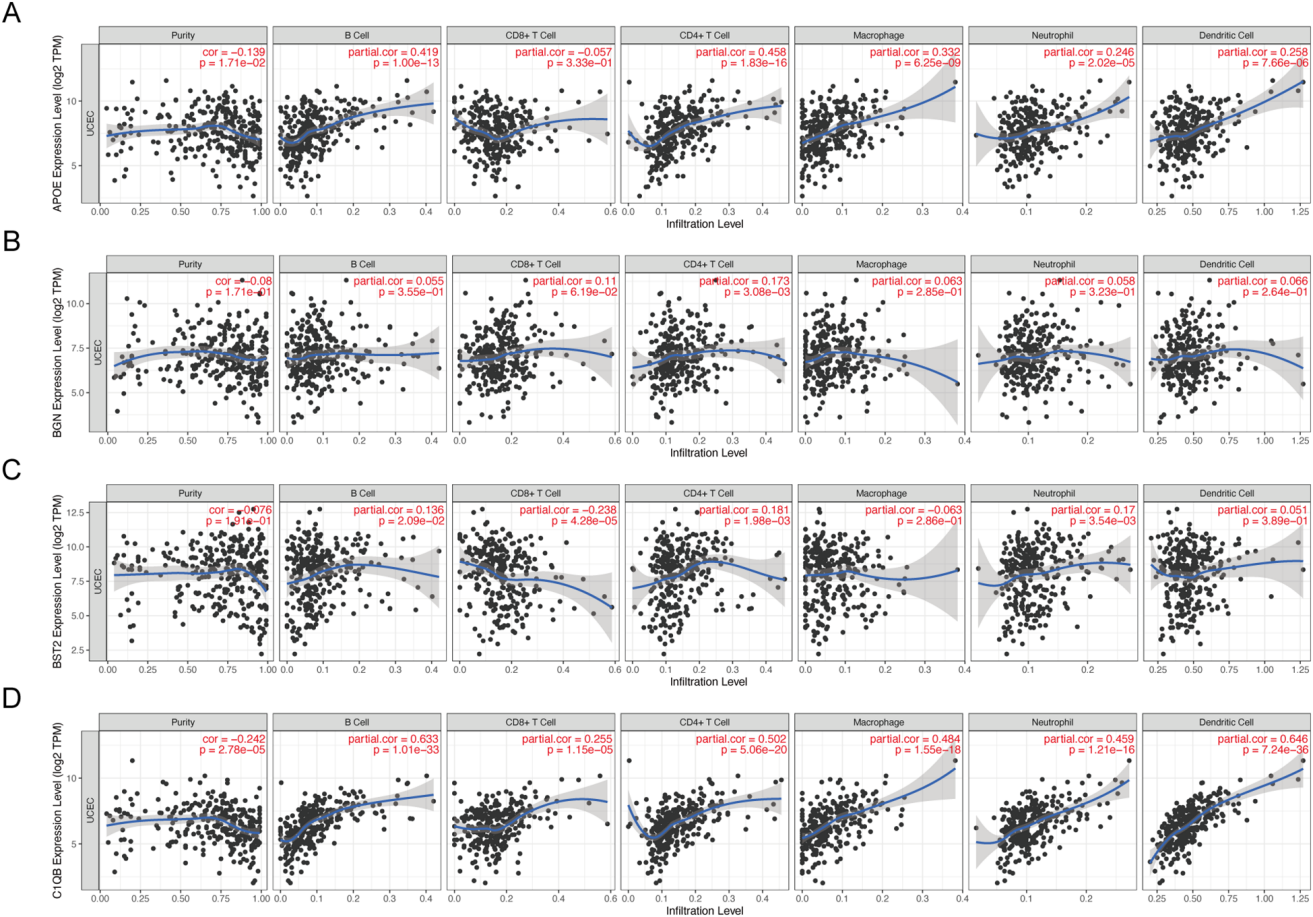
Correlation between hub gene expression and immune cell infiltration level in 4 endometrial carcinomas. APOE **(A)**, BGN **(B)**, BST2 **(C)**, and C1QB **(D)** expression correlated with tumor purity and infiltration levels of B cells, CD8+T cells, CD4+T cells, macrophages, neutrophils, and dendritic cells in endometrial cancer.

The TIMER database was used to analyze the correlation between mRNA expression of APOE, BGN, BST2, and C1QB and infiltrating immune cells in cancer tissues. APOE was correlated with the abundance of B cells (cor=0.419, P=1.00e−13), CD4+ T cells (cor=0.458, P=1.86e−16), macrophages (cor=0.332, P=6.25e−09), and neutrophils (cor=0.246, P=0.005). Dendritic cell abundance (cor=0.258, P=7.66e−06) also showed significant correlation (**Figure 7A**). BGN showed a significant correlation with the abundance of CD4+ T cells (cor=0.173, P=3.08e−03) (**Figure 7B**). BST2 was significantly correlated with the abundance of B cells (cor=0.136, P=2.09e−02), CD8+ T cells (cor=-0.238, P=4.28e−05), CD4+ T cells (cor=0.181, P=1.98e−03), neutrophils (cor=0.17, P=0.017), and CD4+ T cells (cor=0.17, P=0.016) (**Figure 7C**). C1QB was significantly correlated with B cells (cor=0.633, P=1.10e−33), CD8+ T cells (cor=0.255, P=1.15e−05), CD4+ T cells (cor=0.502, P=5.06e−20), macrophages (cor=0.464, P=0.005), neutrophils (cor=0.459, P=1.21e−16), and dendritic cells (cor=0.624, P=7.24e−36). These results provide strong evidence that APOE, BGN, BST2, and C1QB play crucial roles in infiltrating immune cells, including B cells, CD8+ T cells, CD4+ T cells, macrophages, neutrophils, and dendritic cells.

### Candidate drug identification

We entered the 10 most significantly expressed DEGs into the DGIdb database to identify drug-gene interactions and potential drug targets. APOE was associated with 17 drugs for treating endometriosis, ICAM1 with 5 drugs, and MME with 8 drugs for treating endometriosis (**Table 5**). The potential applicability of these drugs as targets awaits further investigation through future cellular and animal studies.

**Table 5:** DEGs-drug interactions in the DGIdb database.

Gene	Drug	Score	Types	Sources	PMIDs
APOE	TROGLITAZONE SOYBEAN OIL ACENOCOUMAROL PREDNISONE PRAVASTATIN STAUROSPORINE SIMVASTATIN TRIAMCINOLONE ATORVASTATIN LUTEIN LORAZEPAM FLUVASTATIN GANCICLOVIR IRBESARTAN FENOFIBRATE RITONAVIR WARFARIN	0.29 1.45 1.82 0.23 0.31 0.27 0.52 0.32 0.30 3.64 0.33 0.52 1.82 0.73 0.25 0.57 0.30	Definite Definite Definite Definite Definite Definite Definite Definite Definite Definite Definite Definite Definite Definite Definite Definite Definite	NCI NCI PharmGKB NCI PharmGKB NCI PharmGKB NCI PharmGKB NCI NCI PharmGKB NCI NCI PharmGKB PharmGKB PharmGKB	15057551 3021887 29432897 3185288 19667110|17289397 11948667 10736278 9150415 16103896|19667110|20031582|17289397 11413081 15699298 17289397|30363031 16322528 12827021 12042669 17700364|15809899 21923605|29432897
ICAM1	ENLIMOMAB BI-505 ALICAFORSEN HYALURONAN LIFITEGRAST	12.37 18.55 27.73 4.12 8.24	Definite Definite Definite Definite Definite	ChemblInteractions TdgClinicalTrial|ChemblInteractions|TTD TdgClinicalTrial|TTD TdgClinicalTrial DTC|TTD	- - - - -
MME	CANDOXATRIL SACUBITRIL GALLOPAMIL SAMPATRILAT LCZ696 PEPINEMAB ILEPATRIL SLV-334	34.78 7.73 2.58 7.73 7.73 3.86 3.86 3.86	Definite Definite Definite Definite Definite Definite Definite Definite	TdgClinicalTrial|TEND|TTD ChemblInteractions TTD TTD TTD TTD TdgClinicalTrial TTD	- - - - - - -

### *In vitro* validation of qRT-PCR

**Figure 8:**
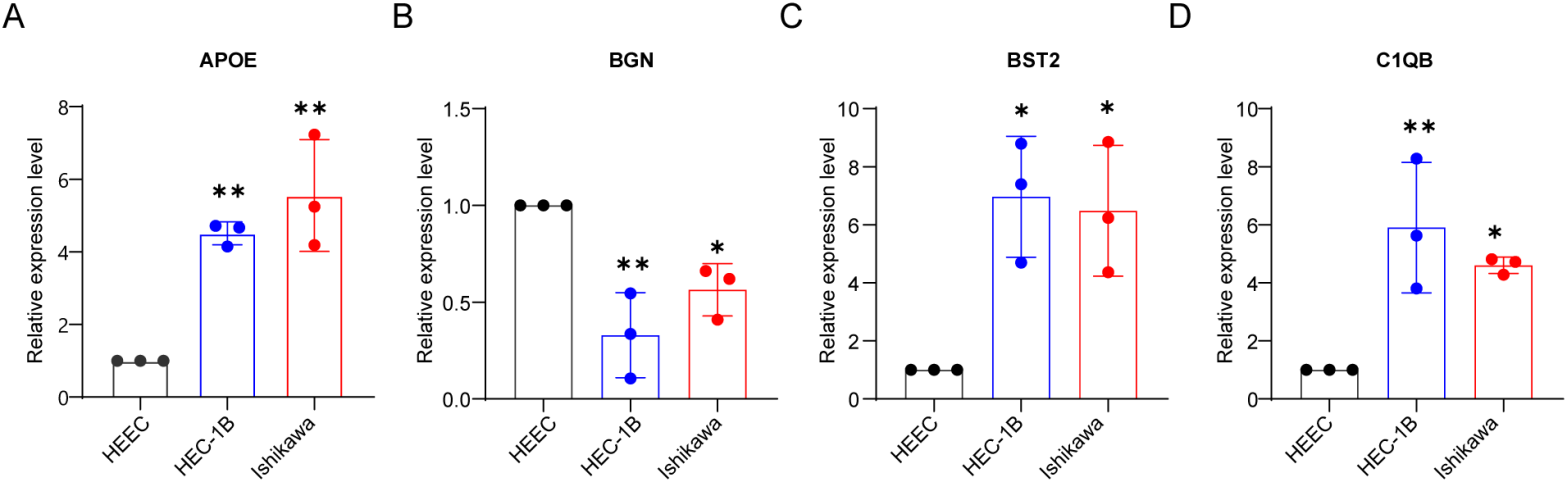
*In vitro* validation of qRT-PCR. Validation of mRNA expression of APOE **(A)**, BGN **(B)**, BST2 **(C)**, and C1QB **(D)** in HEEC, HEC-1B and Ishikawa cell lines by qRT-PCR analysis. *P < 0.05, **P < 0.01.

To further validate our bioinformatics findings, we performed *in vitro* experiments. To further validate our bioinformatics findings, we conducted *in vitro* experiments. Endometrial epithelial cells HEEC were used as a control. We confirmed by qRT-PCR that the mRNA expression levels of APOE, BGN, BST1, and C1QB in endometrial cancer cell lines (HEC-1B and Ishikawa) were consistent with our previous results. The mRNA expression levels of APOE, BST1, and C1QB in endometrial cancer cell lines were significantly higher than those in normal endometrial epithelial cells, whereas BGN expression was higher in normal endometrial cell lines than in endometrial cancer cell lines (**Figures 8A–D**).

## Discussion

Endometriosis is a hormone-dependent chronic inflammatory disease, the exact cause of which remains unclear. It is influenced by retrograde menstruation, coelomic metaplasia, lymphovascular dissemination, as well as genetic and environmental factors (14). Endometriosis is a risk factor for endometrial cancer (13, 15), and the two share common etiological mechanisms, including estrogen stimulation and chronic inflammation (16, 17). Therefore, in this study, we adopted bioinformatics-related methods and utilized the similarities between endometriosis and endometrial cancer to search for targets for the treatment of endometriosis.

By intersecting the datasets of endometriosis and endometrial cancer, we obtained 114 commonly expressed differential genes. Through analyzing the GO functions of DEGs, we found that DEGs were significantly enriched in the regulation of the wnt signaling pathway, growth and development, collagen-related extracellular matrix, and glycosaminoglycan binding. Among them, the abnormality of growth and development-related genes may disrupt the normal physiological functions of endometrial tissue, and the dysregulation of the wnt signaling pathway may lead to endometrial hyperplasia and then trigger endometrial cancer (18). In many solid tumors, due to the massive production of extracellular matrix (ECM) proteins such as collagen by stromal cells, a desmoplastic reaction occurs, resulting in the hardening of the tumor tissue. This fibrotic reaction can inhibit drug delivery and enhance tumor progression and metastasis (19), and fibrotic characteristics have also been found in endometriosis (20, 21). Glycosaminoglycan (GAG) is an important component of the tumor microenvironment (TME). GAG can interact with multiple binding partners, thus affecting cancer progression at multiple levels (22).

Analysis of DEGs in KEGG pathways was conducted to identify the common pathways involved in endometriosis and endometrial cancer. The results showed that the main JAK - STAT signaling pathway and leukocyte transendothelial migration were involved. The inflammation caused by endometriosis is considered to be related to the occurrence of endometrial cancer. The expression of inflammatory mediators may activate signal pathways that promote malignancy in cancer cells and cancer - related inflammatory cells (23, 24). Many inflammatory cytokines transmit their information through the JAK/STAT pathway, which is crucial for immune cell signaling (25). This signaling pathway is involved in the proliferation, migration, and invasion of endometrial cancer cells (2, 26). Recent studies have also shown that the JAK/STAT3 pathway is upregulated in endometriosis and can be used as a treatment target for endometrial cancer (27).

We verified the expression of hub genes through GEPIA and HPA databases. Among them, APOE, BGN, BST1, and BGN showed expression differences in endometrial cancer. Survival analysis indicated that only APOE and BGN were related to the overall survival (OS) and disease-free survival (DFS) of endometrial cancer patients, suggesting that they can be used as biomarkers for predicting patient survival. Additionally, all four genes are involved in regulating cancer cell proliferation, invasion, and migration, which is of great significance in the research of gynecological and obstetrical related tumors. APOE exerts biological functions during certain female fertility disorders (endometriosis) and other gynecological diseases (such as breast cancer, choriocarcinoma, endometrial adenocarcinoma/hyperplasia, and ovarian cancer) (28, 29). The expression level of APOE is related to the histological grade, lymph node metastasis, and FIGO stage in endometrial cancer (30) and stimulates the malignant progression of ovarian cancer by inducing FAK - ERK activation in cell/matrix adhesion (31). BGN enhances the migration and invasion abilities of endometrial cancer (32). Previous studies have found that the expression of BGN is associated with the presence of PTEN loss and KRAS mutation (33, 34). BST2 can promote the metastasis, invasion, and proliferation of oral squamous cell carcinoma through the AKT/ERK1/2 signaling pathway (35). In colorectal cancer, BST2 induces macrophage M2 polarization and promotes tumor progression (36). And C1QB can be used as a potential prognostic biomarker for multiple tumors (37). In cervical cancer, the expression of C1QB protein is related to P16 expression (38). However, their specific relationship with endometrial cancer still needs to be further explored in depth by further studies.

We input the DEGs into the NetworkAnalyst platform to determine the TF gene interactions and TF - miRNA co - regulatory networks. TF genes play a regulatory role according to gene expression, which may lead to the production of cancer cells. From the network, ICAM1 exhibits high interaction rates with other TF-genes, with a degree value of 69 in the TF-genes interaction network. Visualizing the core regulatory network in the TF-miRNA core regulation network helps identify key miRNAs influencing the prognosis of endometriosis. The study identified 70 TF-genes and 37 miRNAs. Among the TFs with the most interactions, ICAM1 has a high degree value of 31, associating with 8 miRNAs. ICAM1 and TIMP1, as regulators of cell adhesion and migration (39), were also highlighted in our analysis. These genes may promote the infiltration and metastasis of tumor cells in the tumor microenvironment by affecting intercellular adhesion and matrix degradation (40–42), similarly in recent years studies have shown that TIMP1 is highly expressed in endometrial cancer and endometriosis tissues (43, 44). Research indicates that ICAM1 can serve as a biomarker for endometriosis (45). Comprehensive analysis of the PPI network, TF-miRNA core regulatory network provides valuable information for understanding the regulatory mechanisms and potential therapeutic targets of endometriosis and its progression.

Tumor-infiltrating immune cells play a crucial role in tumor progression, affecting anticancer treatments and eventually leading to the development of immune tolerance (46). Endometrial cancer is considered an immunogenic disease, and increasing evidence suggests the involvement of the immune system in its progression and patient prognosis (47), Similarly, patients with endometriosis exhibit abnormal distribution of immune cells, and immune system dysregulation holds clinical significance in the pathogenesis (48). Our results showed that the expressions of APOE, BGN, BST1, and BGN were positively correlated with the infiltration status of immune cells such as B cells, CD8 + T cells, and neutrophils in cancer, demonstrating the significance of APOE, BGN, BST1, and BGN in recruiting and regulating infiltrating immune cells in cancer. In our qRT - PCR verification, the expressions of APOE, BGN, BST1, and C1QB in endometrial cancer cell lines were different from those in normal endometrial epithelial cells, which was consistent with our analysis. However, through literature data, we found that BGN may promote the disease progression of most cancers. therefore, there may be some other regulatory relationships between BGN and endometriosis, and we cannot say that it has a certain relationship with the prognosis of endometriosis. Three of the 10 DEGs were found to be associated with drugs in the DGIdb drug database (**Table 5**). The targets of these potentially applicable drugs await further investigation through future cellular and animal studies.

In summary, by integrating data from four databases and employing various bioinformatics methods to explore important gene modules, we identified common genes between endometriosis and endometrial cancer, revealing relative similarities in immune dysfunction between these two diseases. The four hub genes (APOE, BST1, BGN, C1QB) with differential expression in endometrial cancer tissues may serve as potential therapeutic targets, warranting further research to validate their effectiveness as potential prognostic markers and treatment targets for endometrial cancer.

## Data Availability

The raw data supporting the conclusions of this article will be made available by the authors, without undue reservation.
